# [Corrigendum] Effect of overexpression of HOX genes on its invasive tendency in cerebral glioma

**DOI:** 10.3892/ol.2026.15675

**Published:** 2026-06-02

**Authors:** Yun-Bao Guo, Yi-Meng Shao, Jing Chen, Song-Bai Xu, Xing-Dong Zhang, Mao-Ren Wang, Hai-Yan Liu

Oncol Lett 11: 75–80, 2026; DOI: 10.3892/ol.2015.3893

Following the publication of the above paper, it was drawn to the Editor's attention by a concerned reader that, regarding the RT-polymerase chain reaction analyses shown in Fig. 3, the data shown for the *A9* and *D10 HOX* genes were strikingly similar; in addition, the data shown for the *B13* and *D13 HOX* genes were also remarkably similar. Furthermore, the blot for the *A9*/*D10 HOX* genes for the U-118 lane also looked very similar to the blot showing the *D13 HOX* gene in both the U-138 and U-118 lanes, and the data shown for the *A6, A7, D4* and *D9 HOX* genes all bore a strikingly close similarity. Finally, it should be noted that a cursory inspection of the data shown for the RT-polymerase chain reaction analyses in Fig. 2 revealed similar potential anomalies with respect to strikingly similar looking data used to portray various of the *HOX* genes.

These various points were drawn to the authors’ attention courtesy of an expression of concern statement that we published (doi.org/10.3892/ol.2026.15536), and the authors have realized that some of the data were inadvertently selected incorrectly for Figs. 2 and 3. Replacement versions of these figures, now showing alternative data for the RT-polymerase chain reaction analyses, are presented on the next page. Note that the errors made in assembling these figures did not affect the overall conclusions reported in the paper. All the authors agree with the publication of this corrigendum, and are grateful to the Editor of *Oncology Letters* for granting them the opportunity to publish this. Furthermore, they apologize to the readership for any inconvenience caused.

## Figures and Tables

**Figure 2.**
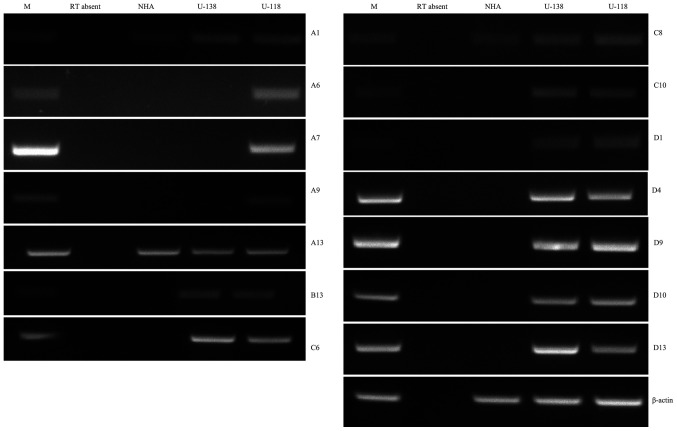
RT-polymerase chain reaction analysis of differentially expressed HOX genes in NHA and U-118 and U-138 glioblastoma multiforme cell lines. Control amplifications of β-actin are also shown. M, normal tissue (negative control without target cDNA); RT, reverse-transcription; NHA, normal human astrocytes.

**Figure 3.**
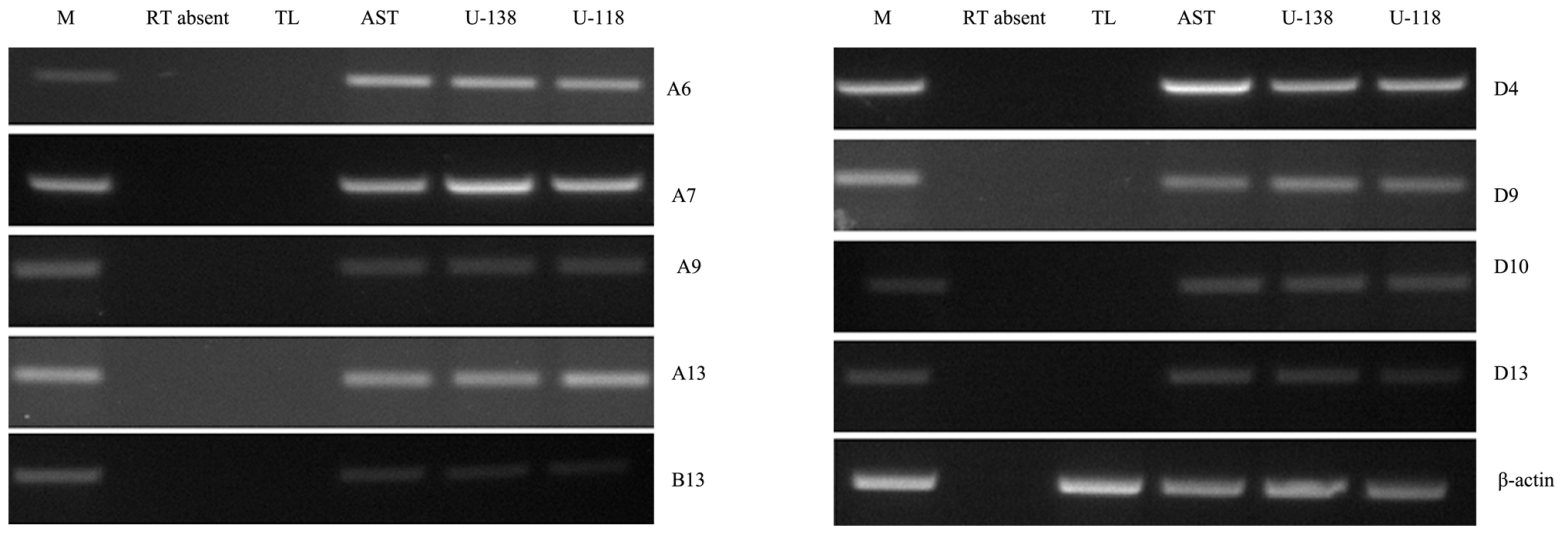
RT-polymerase chain reaction analyses demonstrating differentially expressed HOX genes in normal human TLs, AST samples (grades IV), and glioblastoma multiforme cell lines U-138 and U-118. M, normal tissue (negative control without target cDNA); RT, reverse-transcription; TL, temporal lobe; AST, astrocytoma.

